# A Cytoplasmic Receptor-like Kinase Contributes to Salinity Tolerance

**DOI:** 10.3390/plants9101383

**Published:** 2020-10-17

**Authors:** Nir Sade, Fei Weng, Hiromi Tajima, Yarden Zeron, Lei Zhang, Maria del Mar Rubio Wilhelmi, George Day, Zvi Peleg, Eduardo Blumwald

**Affiliations:** 1 Department of Plant Sciences, University of California, Davis, CA 95616, USA; feiweng91@126.com (F.W.); htajima@ucdavis.edu (H.T.); leizh@ucdavis.edu (L.Z.); mmrubiowilhelmi@ucdavis.edu (M.d.M.R.W.); gday@ucdavis.edu (G.D.); 2 School of Plant Sciences and Food Security, Tel Aviv University, Tel Aviv 69978, Israel; yardenzeron@mail.tau.ac.il; 3 Suzhou Polytechnic Institute of Agriculture, Suzhou 215008, Jiangsu, China; 4 The Robert H. Smith Institute of Plant Sciences and Genetics in Agriculture, The Hebrew University of Jerusalem, Rehovot 7610001, Israel; Zvi.Peleg@mail.huji.ac.il

**Keywords:** receptor-like cytoplasmic kinases, salinity tolerance, *Oryza sativa*, aquaporins

## Abstract

Receptor-like cytoplasmic kinases (RLCKs) are receptor kinases that lack extracellular ligand-binding domains and have emerged as a major class of signaling proteins that regulate plant cellular activities in response to biotic/abiotic stresses and endogenous extracellular signaling molecules. We have identified a rice RLCK (OsRLCK311) that was significantly higher in transgenic pSARK-IPT rice (*Oryza sativa*) that exhibited enhanced growth under saline conditions. Overexpression of OsRLCK311 full-length protein (RLCK311FL) and the C-terminus of OsRLCK311 (ΔN) in Arabidopsis confirmed its role in salinity tolerance, both in seedlings and mature plants. Protein interaction assays indicated that OsRLCK311 and ΔN interacted in-vivo with the plasma membrane AQP AtPIP2;1. The RLCK311-PIP2;1 binding led to alterations in the stomata response to ABA, which was characterized by more open stomata of transgenic plants. Moreover, OsRLCK311-ΔN effect in mediating enhanced plant growth under saline conditions was also observed in the perennial grass *Brachypodium sylvaticum*, confirming its role in both dicots and monocots species. Lastly, OsRLCK311 interacted with the rice OsPIP2;1. We suggest that the rice OsRLCK311 play a role in regulating the plant growth response under saline conditions via the regulation of the stomata response to stress. This role seems to be independent of the RLCK311 kinase activity, since the overexpression of the RLCK311 C-terminus (ΔN), which lacks the kinase full domain, has a similar phenotype to RLCK311FL.

## 1. Introduction

Crop productivity is severely limited by soil salinity. Salinity affects major biosynthetic processes such as photosynthesis, protein synthesis and lipid metabolism [1]. Plant responses to salinity comprise two phases: (i) a fast response to the decrease in soil water potential (more negative) and (ii) a slower response to the toxic effects of Na^+^ ions in the leaves [2]. In general, these responses lead to a strong reduction in stomata conductance and growth retardation [3,4]. While stomata closure will lead to water conservation and enhanced survival to a severe stress, it will also result in a decreased photosynthesis and reduced biomass and yield production [3,5]. Thus, a precise balance between stomata opening, plant growth and active gas exchange needs to be maintained. Under mild stress, plant stress survival responses (i.e., stomata closure and decreased growth) are not necessarily beneficial agronomical traits [3]). The maintenance of a lesser degree of salinity-induced stomata closure might lead to enhanced gas exchange with improved plant biochemical activity and enhanced growth [5,6,7,8,9]. Thus, one might speculate that fine-tuning of the stomata response to salt stress can affect the threshold between water loss, active gas exchange and plant growth. While this phenomenon has been characterized [5,6,7,9], the molecular mechanism(s) regulating it is only beginning to emerge. In recent years, it has been shown that water channels play a role in the regulation of stomata pore opening and gas exchange, particularly under stress conditions [10,11]. The rose RsPIP2;1 was shown to contribute to plant growth strategies under water-deficit stress through alternating between survival (high RsPIP2;1 and low whole leaf transpiration) and biomass accumulation (low RsPIP2;1 and high whole leaf transpiration) [12] (In addition, reverse genetics in maize and Arabidopsis demonstrated that the plasma membrane intrinsic proteins 2 (PIP2) were needed for functional stomata closure under the ABA treatments [13,14]). In maize, *ZmPIP2;5* overexpression and *zmpip2;5* knockout mutant displayed a higher and lower ABA-dependent stomata closure than wild-type plants, respectively (Ding and Chaumont, 2020b). Interestingly, Arabidopsis *atpip2;1* knockout mutant displayed a similar phenotype (i.e., stomata insensitivity to ABA) [13]. Furthermore, the role of AtPIP2;1 in ABA stomata closure was dependent on the AtOST1 protein kinase [13], emphasizing the involvement of kinase-mediated post-translational regulation of AtPIP2;1-dependent stomata closure. Proteomics-based studies showed that AtPIP2;1 could efficiently bind receptor-like kinases (RLK) and receptor-like cytoplasmic kinases (RLCKs), supporting the notion of kinase-mediated PIP2;1 post-translational regulation [15].

Receptor-like cytoplasmic kinases (RLCKs) in plants belong to the super family of receptor-like kinases (RLKs). These proteins are similar to RLKs in their kinase domain but lack an extracellular domain [16]. Based on expression analysis and functional studies, RLCKs have been proposed to play role(s) in diverse biological processes, including the response to biotic and abiotic stress [16]. For example, in rice, 376 RLCKs were identified and about 86 were shown to be responsive to abiotic stress [17]). Based on RLCK expression patterns and their involvement in aquaporin (AQP) channels’ post-translational regulation, we hypothesize that rice RLCKs could be involved in the regulation of the tolerance of rice to salinity stress, possibly through the regulation of stress-dependent stomata closure, plant growth and AQP activity. Here, we identified a single rice RLCK311 (putatively annotated as a serine/threonine-protein kinase BRI1-like 1 precursor) that was upregulated in the response of transgenic *SARK:IPT* rice [18] to salinity. We expressed *RLCK311* in the dicot *Arabidopsis thaliana* and the monocot Brachypodium plants. Our results indicated that the RLCK311 can bind to PIP2;1 in vivo, deactivate PIP2;1-dependent stomata closure and contribute to enhanced plant growth under salinity conditions. Our results indicated that RLCK311-PIP2;1 binding and stomata reliant stress regulation was independent of RLCK311 kinase activity.

## 2. Materials and Methods

### 2.1. Plant Materials and Growth Conditions

Homozygous transgenic T_3_ lines [18] expressing *SARK::IPT* and wild-type (WT) seeds of rice (*Oryza sativa* L. ssp. japonica cv. kitaake) were germinated on moist germination paper (25 × 38 cm; Anchor Paper Co., St. Paul, MN, USA) for 6 days at 30 °C in the dark. Seedlings were then transplanted into 4.5-L pots, filled with soil (Capay series, harvested in California rice fields, 38°32’23.93’’ N,121°48’30.81’’ W, shredded and steamed for 1.5 h to eradicate soil pathogens), with two plants per pot, and placed in water tubes. Plants were grown at 12 h/12 h day/night under an illumination of 1200 µmoL m^−2^s^−1^ at 30 °C⁄20 °C. Plants were fertilized with a solution 50% N:P:K (20:10:20) and 50% ammonium sulphate (total of 0.5 g of N) every 10 days until panicle initiation. Salinity treatments were applied at the panicle initiation stage; NaCl concentrations in the watering solution were increased progressively every 3 days (25, 50, 75 and 100 mM NaCl). A concentration of 100 mM NaCl was maintained for 40 days, until the end of the experiment. *OsRLCK311* expression was measured from RNAseq data obtained from plants grown in the presence of 100 mM NaCl for 3 days (E. Blumwald, unpublished).

Seeds of wild-type and transgenic *Arabidopsis thaliana* (ecotype Columbia) were sterilized and then germinated in 1/2 Murashige and Skoog medium (Sigma-Aldrich, St. Louis, MO, USA) supplemented with 0.5% sucrose. For plate-grown plants, seeds were incubated in medium containing 1% Phytagel ((Sigma-Aldrich, St. Louis, MO, USA)) for 10 days in a growth chamber at 23 ℃ under 100 µmoL m^−2^ s^−1^ light in a 16 h light/8 h dark regime. Seedlings were then transplanted to plates containing 1/2 Murashige and Skoog medium (MS) medium (control) or 1/2 MS medium supplemented with 75 mM NaCl (salt), and plates were incubated as described above in the same growth chamber. Photographs were taken 10 days after transplantation, and seedlings were sampled for fresh weight and malondialdehyde (MDA) measurements.

For soil-grown experiments, seeds of wild-type and transgenic *Arabidopsis thaliana* (Col-0) were grown in autoclaved Sunshine Mix 4 (SunGro, Sacramento, California). Plants were grown in a growth chamber as described above. Salt stress treatments were applied by irrigating with a solution containing 125mM NaCl at 30 days after seed germination. Photographs were taken at 7 days after salt stress was applied, and dry weight of the full rosettes were measured. Fully expanded leaves were sampled to determine the chlorophyll and MDA contents.

Seeds of wild-type *Brachypodium sylvaticum* and two T_3_ homozygous transgenic lines were germinated on moist germination paper for 5 days at 4 °C in the dark. Young seedlings in the paper rolls were then moved to the growth chamber under 100 µmoL m^−2^ s^−1^ light in a 16-h light/8 h-dark at 26 °C/20 °C. Twenty-day-old seedlings were transplanted to autoclaved Sunshine Mix 4 fertilized with Osmocote (ICL, Tel-Aviv, Israel) (N:P:K) (14:14:14) and kept well-watered with distilled water. Salt stress treatment was applied at 30 days after seed germination by irrigating with a 100mM NaCl solution for 7 days, and then increasing the salt concentration to 200 mM NaCl until harvest. Shoot dry weight was measured 2 weeks after the initiation of the stress treatment. Fully expanded leaves were sampled for qRT-PCR analysis. All plants were irrigated with DI water for another 25 days until the second harvest.

### 2.2. Protein Sequences Alignment

Protein sequences of RLCK311 full-length (FL) (LOC_Os11g06780) and RLCK311 C-terminal domain (ΔN) (Os11g0168600) were obtained from rice genome databases (http://rice.plantbiology.msu.edu/ and https://rapdb.dna.affrc.go.jp/). The protein sequences alignment was performed in the plasmid editor ApE.

### 2.3. Generation of Transgenic Plants

All the constructs were generated using the Gateway system (Invitrogen, Carlsbad, CA, USA).

For *35S::RLCK311-ΔN::3×Flag Arabidopsis thaliana* transgenic plants, the ORFs of *OsRLCK311-*ΔN and *OsRLCK311*-FL were amplified from *Oryza sativa* cDNA and cloned into pDONR207 by BP reaction to generate pEN-ΔN and pEN-OsRLCK311FL. Meanwhile, 35S promoter was also amplified from pEarlyeGate101 [19] and cloned into pDONR P4P1R vector (Invitrogen). These two entry clones and pEN-R2-3xFlag-L3 [20] were recombined via multisite LR reaction into pB7m34GW [21], resulting in pB7m34GW-35S:ΔN /OsRLCK311FL:3XFlag.

For transgenic *Brachypodium sylvaticum* plants expressing *pSARK:OsRLCK311300*, the sequence of *SARK* promoter was amplified and cloned into pDONR P4P1R vector by BP reaction to generate pEN-pSARK. The pEN-ΔN and the pEN-pSARK were recombined via multisite LR reaction into pH7m24GW,3 [21], resulting in pH7m24GW-pSARK:ΔN.

For transgenic *Brachypodium sylvaticum* plants expressing *pSARK::GUS(+)*, GUSplus gene was amplified from pCAMBIA1305.2 and cloned into pDONR207 by BP reaction to generate pEN-GUS+. Gateway sequence fragment was amplified from pH7m24GW and inserted into pZH2B (Kuroda et al., 2010) to accommodate two fragment multisite gateways in a 35S:Hyg vector for monocot transformation as pZH2B-GW. pEN-pSARK and pEN-GUS+ were recombined via multisite LR reaction into pZH2B-GW, resulting in pSARK:Gus(+).

### 2.4. Malondialdehyde Measurements

Young seedlings or fully expanded leaves were weighed and then ground in liquid N_2_. The tissue was homogenized in the presence of 10% trichloroacetic acid containing 0.25% thiobarbituric acid. The mixture was heated at 95 °C for 30 min, quickly cooled in ice, and then centrifuged at 10,000 xg for 10 min. The absorbance of the supernatant was measured at 532 nm, 600 nm and 450 nm (Synergy^TM^ Mx Microplate Reader; BioTek, Winooski, VT, USA). The concentration was calculated according to the equation C (μmol/L) = 6.45 × (A532 − A600) − 0.56 × A450 [22].

### 2.5. Chlorophyll Measurements

Fully expanded leaves were ground in liquid N_2,_ and chlorophyll was extracted in 80% acetone. Absorbance at 663 nm and 645 nm was measured (Synergy^TM^ Mx Microplate Reader; BioTek, Winooski, VT, USA), and chlorophyll contents were calculated as described in [23].

### 2.6. Protein Interaction Assays

For RLCK311-ΔN, OsRLCK311-FL and AtPIP2;1 colocalization assays, the ORFs of *RLCK311-ΔN* and *OsRLCK311-FL*, excluding the stop codon, were amplified from rice leaf cDNA and cloned into pDONR207 by BP reaction. pEN-ΔN or pEN-OsRLCK311FL was recombined via LR reaction into the destination vector pEarleyGate 103 [19] for green fluorescent protein (GFP)fusion. The genes *AtPIP2;1, OsPIP2;1* and *OsBIN2* were fused with RFP of another destination vector pH7RWG2 using the same strategy as described above. The resulting constructs were introduced into *Agrobacterium tumefaciens* GV3101 and co-infiltrated into the abaxial surface of the leaves of 4-week-old *Nicotiana benthamiana* as described previously [24]. Fluorescence microscopy was performed two d after infiltration with an inverted Zeiss LSM 710 confocal laser scanning microscope (Carl Zeiss, Oberkochen, Germany) equipped with a 20× water immersion objective. The excitation wavelength/emission were as follows: GFP (488 nm/500–530 nm) and red fluorescent protein (RFP, 561 nm/600–660 nm).

For bimolecular fluorescence complementation (BiFC), the vectors pDEST-^GW^VYNE and pDEST-^GW^SCYCE from the Gateway-based BiFC vector systems [25] were employed to fuse OsRLCK311300 and OsRLCK311FL with the N-terminus of yellow fluorescent protein Venus (Venus^N^) and fused to AtPIP2;1, OsPIP2;1 and OsBIN2 with the C-terminus of super CFP (SCFP^C^) to obtain the constructs ΔN /OsRLCK311FL-Venus^N^ and AtPIP2;1/OsPIP2;1/OsBIN2-SCFP^C^. All the constructs were introduced into *Agrobacterium tumefaciens* GV3101. Transient expression and fluorescence microscopy were performed at the same conditions as described for protein colocalization.

For co-immunoprecipitation and mass spectrometry analysis, three-week-old seedlings of wild-type and transgenic plants overexpressing *OsRLCK311-ΔN* /*OsRLCK311-FL* were ground in liquid N_2_, and incubated on an end-over-end rotator at 4 °C for 4 h with lysis buffer provided in the μMACS Isolation Kit (Miltenyl Biotec, Bergisch Gladbach, Germany), containing protease inhibitor cocktail (Sigma-Aldrich, St. Louis, MO, USA) and 1 mM PMSF. The mixture was centrifuged at 10,000×g for 10 min at 4 °C, and the supernatant was used for co-immunoprecipitation (COIP). COIP was performed using anti-Flag magnetic beads according to the manufacturer protocol (Pierce™ Anti-DYKDDDDK Magnetic Agarose, Invitrogen, USA). LC-MS/MS analysis was performed at the Proteomics Core Facility of the University of California, Davis, as described previously [26]. Scaffold (version Scaffold 4; www.proteomesoftware.com) was used to validate tandem MS-based peptide and protein identification. The results were filtered with peptide thresholds (63% minimum) and protein thresholds (80% minimum), and two unique peptide required for identification. Although the filtration thresholds used in our study are somewhat low, they allowed for the identification of proteins forming complexes through binding both FL and ΔN-OsRLCK311. The putative interactions were further confirmed by BiFC assays (see above) and co-immunoprecipitation. Full interactors peptide and samples report is presented in Tables S3 and S4.

For co-immunoprecipitation by transient expression and western blots, OsRLCK31-ΔN-GFP/OsRLCK311FL-GFP was co-infiltrated with AtPIP2;1-RFP, OsPIP2;1-RFP or free RFP as described above. Samples displaying protein expression as measured by fluorescence microscopy (Figures S5 and S6) were used 2 daysafter infiltration. Leaf tissue was grinded in liquid N_2,_ and lysis buffer (as described above) was added (2 mL/g fresh weight). Following incubation and centrifugation, 1 mL of the supernatants was purified through anti-GFP magnetic beads from the μMACS GFP Isolation Kit (Miltenyl Biotec, Bergisch Gladbach, Germany) and applied for immunoblot analysis using anti-GFP (NB600-308SS, Novud Biologicals, Centennial, CO, USA) and anti-RFP antibody (NBP2-25157SS, Novus biologicals, Colorado, USA) with a 1:1000 or 1:500 dilution, respectively.

### 2.7. GUS Staining

Seeds of wild-type *Brachypodium sylvaticum* and two transgenic lines were germinated on moist germination paper for 5 days at 4 °C in the dark. Young seedlings were then moved from the paper rolls to the growth chamber and grown at 26 °C/20 °C (day/night) 100 µmol m^−2^ s^−1^ light in 16h/8h day/night regime. Paper rolls with 20 d-old seedlings were exposed to 200mM NaCl for 24 h and stained using standard GUS protocols [27].

### 2.8. Quantification of Stomata Aperture

The epidermis of fully expanded leaves was carefully peeled from the abaxial surface and immediately incubated in MES-KCl buffer (5 mM KCl/10 mM Mes/ 50 µM CaCl2, pH 6.15) under 100 µmol m^−2^ s^−1^ light for 2 h. After 2 h, 50 µM ABA in DMSO was added to the MES-KCl buffer. Epidermis peels and buffers were mixed by gentle shaking for 20 min and kept under 100 µmol m^−2^ s^−1^ light all the time. Following incubation, stomata were observed using a fluorescence digital microscope (Olympus, Tokyo, Japan) and stomata aperture was analyzed by Image J.

## 3. Results

### 3.1. Overexpression of OsRLCK311 and Its C Terminus Domain (ΔN) in Arabidopsis Conferred Salt Tolerance

RLCKs, which lack extracellular ligand binding domains, are associated with signalling and many cellular processes [16], and a number of RLCKs were shown to be associated with the response(s) of rice to abiotic stress [17]. *OsRLCK311*, a gene encoding a Receptor-Like Cytoplasmic Kinase (RLCK), one of these proteins, was highly expressed in transgenic rice plants overexpressing *IPT*, a gene encoding ISOPENTENYL TRANSFERASE (regulating cytokinin synthesis), driven by *P_SARK,_* a stress-induced promoter. The expression of *P_SARK_:IPT* resulted in delayed stress-induced senescence and increased abiotic stress tolerance of the transgenic rice plants ([18]; Figure S1). *OsRLCK311*, annotated as a brassinosteroid-insensitive like precursor (BRIL) in the rice genome database (http://rice.plantbiology.msu.edu/), comprises 389 amino acids. It has been also annotated as a shorter version with 91 amino acids in a different rice genome database (https://rapdb.dna.affrc.go.jp/). Protein sequence alignment revealed that the shorter version is similar to the C-terminus domain (ΔN) of the full-length protein (FL) (Figure S2).

**Figure 1:**
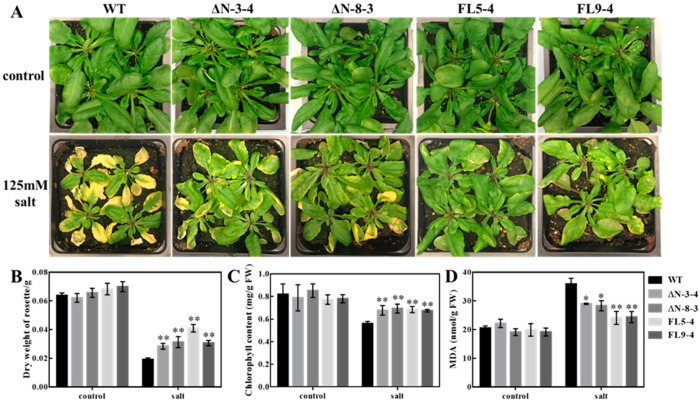
Salt stress tolerance of wild-type (WT) and RLCK311 over-expressing lines grown in soil. (**A**) Growth of wild-type (WT) and different *RLCK311* over-expressing lines under normal condition (control) or salt stress (treated with 125mM NaCl for 7 days). (**B**) Dry weight of each rosette, (**C**) chlorophyll content and (**D**) malondialdehyde (MDA) content of wild-type (WT) and all RLCK311 over-expressing plants. Values represent means ± SE (*n* = 12). Asterisks represent significant differences between transgenic lines and wild-type within a treatment by the Dunnet test (*, *p* < 0.05 and **, *p* < 0.01).

In order to evaluate the roles of OsRLCK311 in salinity stress responses, the full-length *OsRLCK311* (FL) and the *OsRLCK311* C-terminus (ΔN) were overexpressed and transgenic lines were generated in Arabidopsis (Figure S3). When grown under salinity conditions, FL- and ΔN-overexpressing Arabidopsis plants displayed enhanced growth, higher dry weight and lower MDA contents than wild-type plants (Figure 1 and Figure S4).

### 3.2. OsRLCK311 Binds to Aquaporin AtPIP2;1

**Figure 2:**
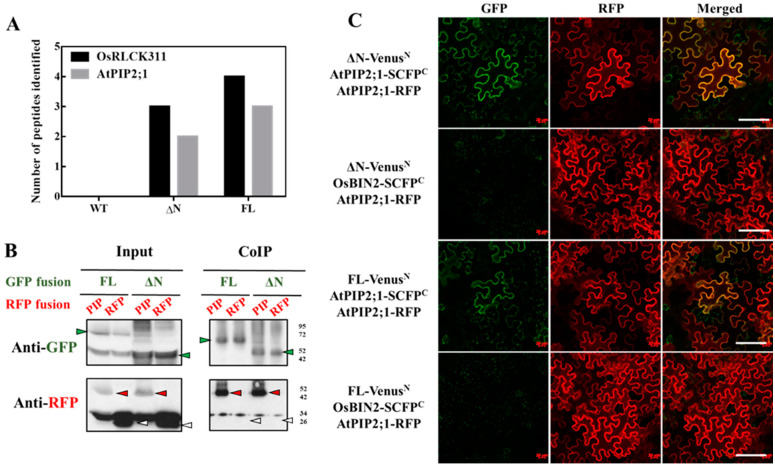
Rice RLCK (OsRLCK311) interacts with an aquaporin protein AtPIP2;1 in vivo. (**A**) AtPIP2;1 interacts with both ΔN and full-length (FL) through co-immunoprecipitation and identified by MS. (**B**) Co-immunoprecipitation of ΔN or FL with AtPIP2;1 by transient expression in *Nicotiana benthamiana*, identified by western blots. The proteins extracted from tobacco leaves infiltrated with both ΔN-GFP/FL-GFP (green arrows) and AtPIP2;1-RFP (red arrows) were co-immunoprecipitated (IP) with anti-GFP antibodies and blotted with anti-GFP or anti-red fluorescent protein (RFP) antibodies. Free RFP is marked in white arrows. ‘’Input’’ stands for protein extracts before immunoprepicitaton. (**C**) Bimolecular fluorescence complementation (BiFC) analysis of the interaction between ΔN or FL with AtPIP2;1 by transient expression in *Nicotiana benthamiana*. Venus^N^ was fused at the C-terminus of ΔN and FL, while SCFP^C^ was fused to the C-terminus of AtPIP2;1 and OsBIN2. Infiltration with OsBIN2-SCFP^C^ were used as negative controls. AtPIP2;1-mcherry was used as a plasma membrane marker. Bar = 200 μM.

**Figure 3:**
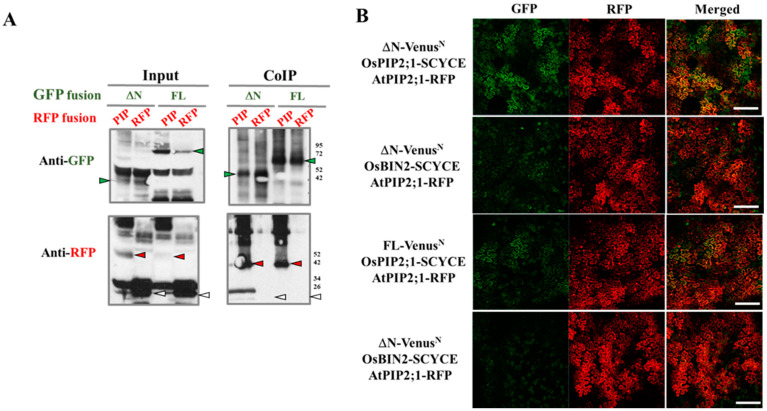
OsRLCK311 interacts with an aquaporin protein OsPIP2;1 in vivo. (**A**) Co-immunoprecipitation of ΔN or FL with OsPIP2;1 by transient expression in *Nicotiana benthamiana* and then identified by western blot. The proteins extracted from tobacco leaves infiltrated with both ΔN-GFP/FL-GFP (green arrows) and OsPIP2;1-RFP (red arrows) were co-immunoprecipitated (IP) with anti-GFP antibody and blotted with anti-GFP or anti-RFP antibody. Free RFP is marked in white arrows. ‘’Input’’ stands for protein extracts before immunoprepicitaton. (**B**) BiFC analysis of the interaction between ΔN or FL with OsPIP2;1 by transient expression in *Nicotiana benthamiana*. VenusN was fused at the C-terminus of ΔN and FL, while SCFPC was fused to the C-terminus of OsPIP2;1 and OsBIN2. Infiltration with OsBIN2-SCFPC were used as negative controls. AtPIP2;1-mcherry was used as a plasma membrane marker. Bar = 200 μM.

To reveal possible mechanisms mediating OsRLCK311 function(s) in the response to salinity, proteins interacting with OsRLCK311-FL and its C-terminus (ΔN) were identified by immunoprecipitation and subsequent mass spectrometry. Immunoprecipitated ΔN/FL–3×Flag and its interacting proteins were obtained from total protein extracts from 3-week-old seedlings of wild-type and transgenic plants overexpressing *OsRLCK311FL/*ΔN. Protein extracts from wild-type plants were used as a negative control to exclude the proteins non-specifically binding to the anti-Flag magnetic beads. A number of candidate proteins were identified (Table S2), among them Aquaporin AtPIP2;1 (Figure 2A). Co-immunoprecipitation by *OsRLCK311ΔN/FL* transient expression of tobacco leaves and western blots confirmed the interaction between OsRLCK311ΔN/FL and AtPIP2;1 (Figure 2). Full-length OsRLCK311-FL fused with GFP (FL-GFP) or OsRLCK311- ΔN fused with GFP (ΔN-GFP) were co-infiltrated with AtPIP2;1-RFP, and leaves displaying high GFP and RFP abundance were used for immunoprecipitation (Figure S5). AtPIP2;1-RFP, but not free RFP, was immunoprecipitated by ΔN/FL-OsRLCK311-GFP (Figure 2B). BiFC was used to confirm the interaction. The transient expression of fusion proteins ΔN/FL-Venus^N^ and AtPIP2;1-SCFP^C^ in *N. benthamiana* resulted in BiFC fluorescence, which co-localized with the plasma membrane marker AtPIP2;1-RFP (Figure 2C). Similar results were obtained when *OsRLCK311* and *Os OsPIP2;1* were transiently expressed in tobacco leaves. CoIP and western blots showed that OsPIP2;1-RFP was immunoprecipitated by ΔN/FL-GFP (Figure 3 and Figure S6), and that the interaction occurred at the plasma membrane.

### 3.3. OsRLCK311 Negatively Regulates Stomata Response to ABA 

**Figure 4:**
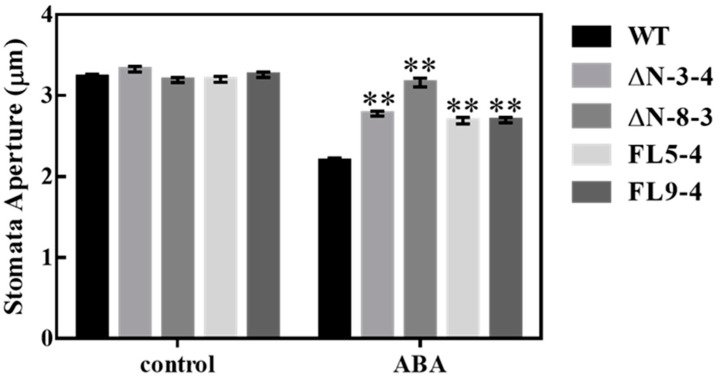
Epidermal strips of wild-type (WT) and all RLCK311 over-expressing lines were first incubated in stomatal opening buffer under the light and then treated with 0 or 50 μM ABA for 20 min, and stomatal pore area was microscopically assessed. Values plots of > 700 measurements (stomata). Asterisks represent significant differences between transgenic lines and wild-type within a treatment by the Dunnet test (*, *p* < 0.05 and **, *p* < 0.01).

AtPIP2;1 knockout resulted in the loss of ABA-induced stomata closure [13]. In order to assess whether the OsRLCK311-AtPIP2;1 interaction led to the deactivation of AtPIP2;1-mediated stomata response to ABA, stomatal aperture was measured in epidermal peels of wild-type and transgenic plants over-expressing ΔN/FL. Over-expression of ΔN/FL significantly suppressed ABA-mediated stomatal closure (Figure 4). Notably, the expression of both *OsRLCK311-FL* and *OsRLCK311- ΔN* suppressed ABA-mediated stomatal closure, suggesting that the action of OsRLCK311 was independent of its kinase activity.

### 3.4. Salinity-Induced Expression of OsRLCK311-ΔN Conferred Salt Tolerance in the Perennial Grass Brachypodium sylvaticum

**Figure 5:**
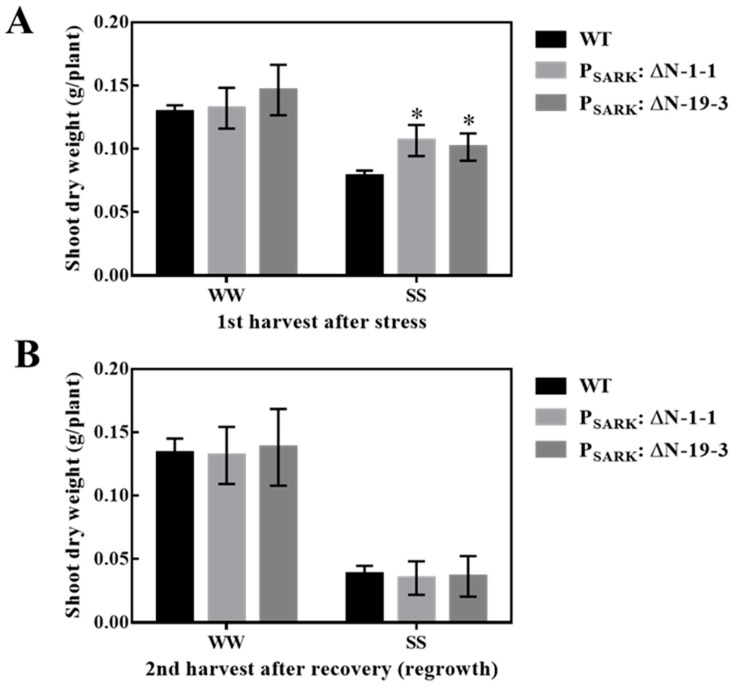
Salt stress tolerance of wild-type (WT) and transgenic *Brachypodium sylvaticum* plants expressing pSARK: ΔN. (**A**) Shoot dry weight of WT and two transgenic lines (SARK: ΔN-1-1 and SARK: ΔN-19-3) grown under well-watered (WW) and salt stress (SS) conditions during the first harvest. Plants were harvested for the first time at around 45 days old, right after being treated with 100mM NaCl solution for 1 week, and then 200mM NaCl solution for 1 week continuously. (**B**) Shoot dry weight of wild-type (WT) and two transgenic lines during the second harvest. The second harvest was performed at 25 days after the first harvest, during which all the plants were well irrigated with distilled water. Values are means and SE (*n*= 12 for SARK:ΔN and 48 for WT). Asterisks represent significant differences between transgenic lines and WT within a treatment by the Dunnet test (*p* < 0.05).

OsRLCK311-ΔN, driven by the stress-inducible promoter SARK, was introduced into the perennial grass *Brachypodium sylvaticum* (Figure S7). Transgenic *P_SARK_::RLCK311-ΔN B. sylvaticum* plants displayed enhanced growth under salt stress (Figure 5A). The shoot dry weight of transgenic plants was higher than that of wild-type plants at the first harvest after stress (Figure 5A). Since *B. sylvaticum* is a perennial grass, the re-growth capacity was assessed by a 2nd harvest following recovery under well-watered conditions. No significant difference was observed between genotypes, indicating no negative effect on perenniality (Figure 5B).

## 4. Discussion 

RLCKs have been shown to be important players in plant receptor kinase-mediated signalling [16]. *OsRLCK311* expression was correlated with the enhanced stress tolerance of transgenic rice plants, brought about delayed stress-induced senescence [18], and the overexpression of *OsRLCK311* in Arabidopsis plants resulted in enhanced growth under saline conditions (Figure 1). These results agreed with observations showing that soybean RLCKs negatively regulated ABA signalling and contributed to increased abiotic stress tolerance in Arabidopsis transgenic plants expressing *GsRLCKs* [28,29]. 

OsRLCK311 interacted with AtPIP2;1 (Figure 2). Aquaporins have been shown to regulate the stomata aperture under stress-like conditions [10,11,13,14] and to regulate the trade-off between plant survival and plant growth (i.e., water conservation versus growth/gas exchange) [12]. Plants overexpressing *OsRLCK311* displayed an altered response to ABA, leading to increased stomata opening, possibly due to the deactivation of PIP2;1 [13]. RLCK311 also possibly interacted with a GRF 14-3-3 protein (Figure S8). 14-3-3 proteins have been shown to interact with water channels [30], and more specifically, with Arabidopsis AtPIP2;1, to regulate oscillations in water homeostasis [31]. Our results suggest that RLCK311 forms a complex with PIP2;1 and a 14-3-3 protein, regulating ABA-dependent stomata response. Moreover, phospholipase D-alpha 1, which is involved in ABA-dependent stomata regulation [32], was also identified as potential interactor with RLCK311 (Table S2), supporting the notion of a role of RLCK311 in the response of stomata to ABA. Interestingly, the transgenic plants overexpressing *OsRLCK311-ΔN* also displayed enhanced salinity tolerance (Figure 1). Moreover, the contribution of OsRLCK311-ΔN to salinity tolerance was also observed in the perennial grass *Brachypodium sylvaticum* (Figure 5). Since in-silico analysis and autophosphorylation assays showed no active kinase domain in OsRLCK-ΔN (not shown), our results suggest that the role(s) of OsRLCK311 in stress tolerance was independent of this kinase activity. Research is needed to assess the possibility that OsRLCK311 may function as a scaffold protein, bringing together cytoplasmic signalling components affecting aquaporin function(s).

Depending on the type and severity of the stress, the overall environmental conditions, and the plant species, plant response(s) to stress include, avoidance, escape or tolerance strategies [33]. For example, an avoidance strategy, which includes decreased stomata conductance and plant growth, could be beneficial for plant survival under severe stress, but not necessarily as a response to a mild stress [3] where the maintenance of an active gas exchange could contribute to plant growth and metabolism [9,34,35,36]. Moreover, under low vapour pressure conditions (e.g., high humidity) where transpiration flow is relatively low, open stomata would allow continuous CO_2_ diffusion with minimum water loss. OsRLCK311 could contribute to determining a stomata threshold response to environment conditions via its interactions with stomata response proteins. Although OsRLCK311 binding to PIP2;1 led to adjustment in stomata closure, similar interactions in other tissues (e.g., roots, mesophyll, xylem parenchyma) cannot be excluded. In rice, OsPIP2;1 was shown to regulate root hydraulic conductivity [37] while Arabidopsis AtPIP2;1 was shown to regulate leaf hydraulic conductivity via xylem parenchyma [38]. In addition, since OsRLCK311 has been identified in transgenic plants with modified cytokinin production, it would be interesting to further test the link between OsRLCK311-mediated stress tolerance and cytokinin response.

## Supplementary Materials

The following are available online at https://www.mdpi.com/2223-7747/9/10/1383/s1, Figure S1: Rice expressing PSARK::IPT showing salinity stress tolerance, Figure S2: Alignment of RLCK311 full length protein FL (LOC_Os11g0678) and RLCK311 C terminal ΔN (Os11g0168600) from the two rice genome databases, Figure S3: Expression of OsRLCK311 (ΔN or FL) in transgenic Arabidopsis plants. qRT-PCR analysis was conducted by using 30-day-old plants grown under normal conditions, Figure S4: Salt stress tolerance of 20 days-old Arabidopsis wild type (WT) and RLCK311 over-expressing lines grown in MS plates with and without 75 mM NaCl for 10 days, Figure S5: Infiltrated leaves taken for blots shown in Figure 2(B) The entire blots of Figure 2, Figure S6: Infiltrated leaves taken for blots shown in Figure 3(B). The entire blots of Figure 3. Figure S7: Gene expression driven by a stress and maturation induced promoter (SARK) promoter. Figure S8:MS identification of GRF9 and GRF10 interacting with both RCLK311-ΔN and RCLK311-FL, Table S1: List of the primers used in the experiments, Table S2: List of candidates RLCK311 interactors, Table S3: list of all MS interactors samples, Table S4: list of all peptide interactors.
