# Supplementary material for: A Cytoplasmic Receptor-like Kinase Contributes to Salinity Tolerance
Source: Plants (Basel). 2020 Oct 17;9(10):1383. doi: 10.3390/plants9101383 (PMC7650656; doi:10.3390/plants9101383)
Supplement: Supplementary file 1 [file plants-09-01383-s001.zip › Supplemental figure legends.docx]

Fig. S1. Rice expressing PSARK::IPT showing salinity stress tolerance

(A) Fresh weight of WT and PSARK::IPT lines (5T and 8T) under well water (WW) and salinity stress (SS) and (B) expression (FPKM) of RLCK311 full length (LOC_Os11g0678) in PSARK::IPT line 5T under WW and SS conditions. Asterisks represent significant differences between transgenic lines and WT within a treatment by the Dunnet test (*, P<0.05).

Fig. S2 Alignment of RLCK311 full length protein FL (LOC_Os11g0678) and RLCK311 C terminal ΔN (Os11g0168600) from the two rice genome databases (http://rice.plantbiology.msu.edu/ and https://rapdb.dna.affrc.go.jp/, respectively)

Fig. S3 Expression of OsRLCK311 (ΔN or FL) in transgenic Arabidopsis plants. qRT-PCR analysis was conducted by using 30-day-old plants grown under normal conditions.

Fig. S4. Salt stress tolerance of 20 days-old Arabidopsis wild type (WT) and RLCK311 over-expressing lines grown in MS plates with and without 75 mM NaCl for 10 days. (A) Fresh weight of wild type (WT) and FL over-expressing plants (FL5-4 and FL9-4). (B) Malondialdehyde (MDA) content of wild type (WT) and FL over-expressing plants (FL5-4 and FL9-4). (C) Fresh weight of wild type (WT) and ΔN over-expressing plants (ΔN-3-4 and ΔN-8-3). (D) Malondialdehyde (MDA) content of wild type (WT) and ΔN over-expressing plants (ΔN-3-4 and ΔN-8-3). Values are means and SE (n=12). Asterisks represent significant differences between transgenic lines and WT within a treatment by the Dunnet test (*, P<0.05 and **, P<0.01).

Fig. S5. (A) Infiltrated leaves taken for blots shown in Fig 2(B) The entire blots of figure 2B. Full length of RLCK311 (FL) or short RLCK311 (ΔN) fused with GFP were co-infiltrated with PIP2;1-RFP (PIP) or free RFP (RFP). Protein extracts (Input) were mixed with GFP magnetic beads to isolate GFP fusion proteins and those interactors as coimmunoprecipitates (CoIP). Western blot has been done with anti-GFP (top panels) and anti-RFP (bottom panels) antibodies. Arrowheads show the position of GFP and RFP fusion proteins in GFP and RFP antibody western blots, respectively.

Fig. S6. (A) Infiltrated leaves taken for blots shown in Fig 3 (B) The entire blots of figure 3A. Full length of RLCK311 (FL) or short RLCK311 (ΔN) fused with GFP were co-infiltrated with PIP2;1-RFP (PIP) or free RFP (RFP). Protein extracts (Input) were mixed with GFP magnetic beads to isolate GFP fusion proteins and interacting proteins those interactors as coimmunoprecipitates (CoIP). Western blot were performed with anti-GFP (top panels) and anti-RFP (bottom panels) antibodies. Arrowheads show the position of GFP and RFP fusion proteins in GFP and RFP antibody western blots, respectively.

Fig. S7 Gene expression driven by a stress and maturation induced promoter (SARK) promoter. (A) GUS plus staining of transgenic Brachypodium Sylvaticum plants expressing pSARK:GUS(+) grown under control and salt stress conditions. Leaves were sampled from 2-week old seedlings grown in paper rolls. (B) Expression of ΔN in transgenic Brachypodium Sylvaticum plants expressing pSARK: ΔN grown under control and salt stress conditions. qRT-PCR analysis was performed by using mature leaves before the first harvest.

Fig. S8 MS identification of GRF9 and GRF10 interacting with both RCLK311-ΔN and RCLK311-FL.
