# Supplementary material for: A Cytoplasmic Receptor-like Kinase Contributes to Salinity Tolerance
Source: Plants (Basel). 2020 Oct 17;9(10):1383. doi: 10.3390/plants9101383 (PMC7650656; doi:10.3390/plants9101383)
Supplement: Supplementary file 1 [file plants-09-01383-s001.zip › Supplementary Figures.pptx]

## Slide 1
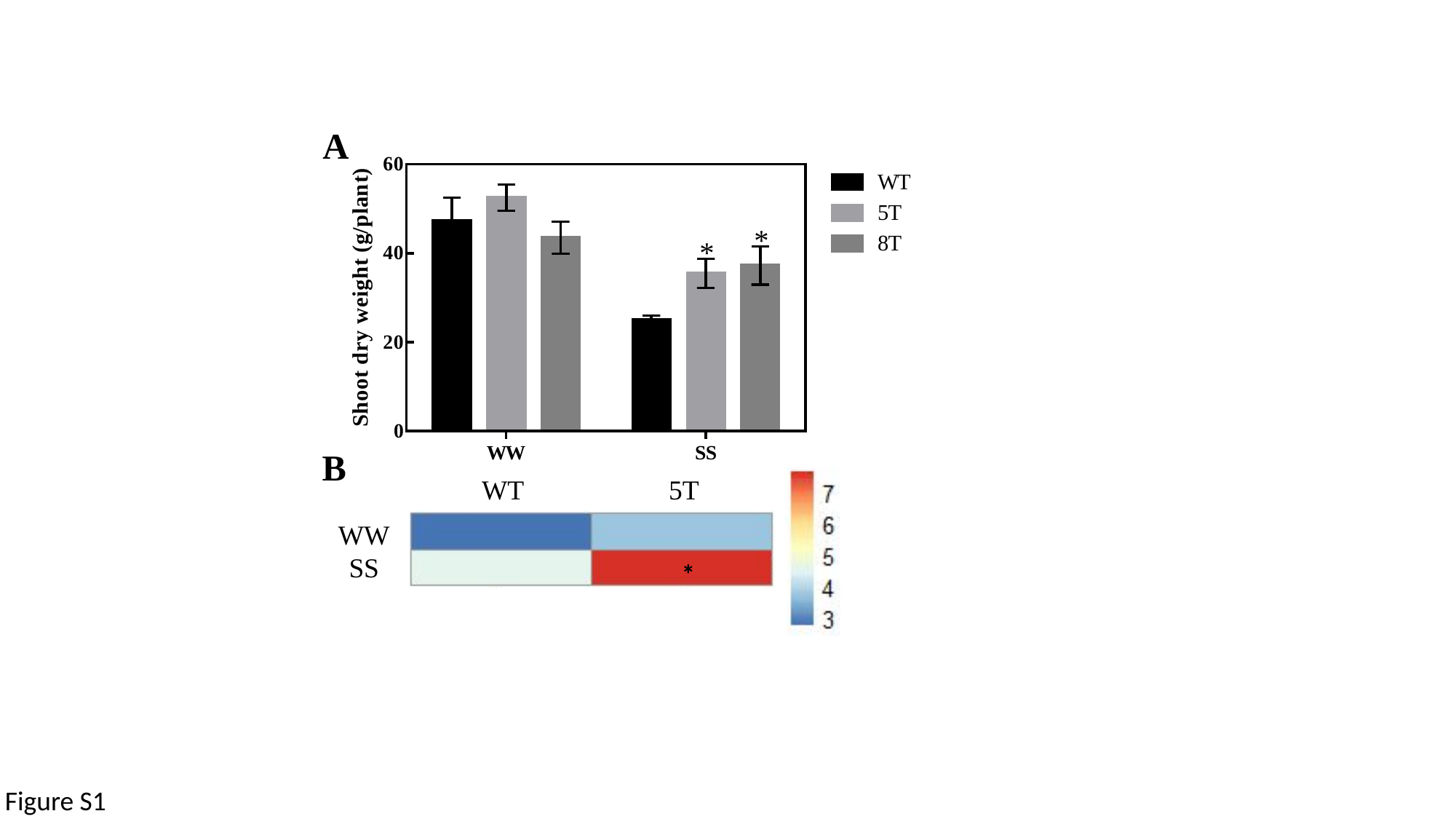

A
B
WT 5T
WW
SS
*
Figure S1

## Slide 2
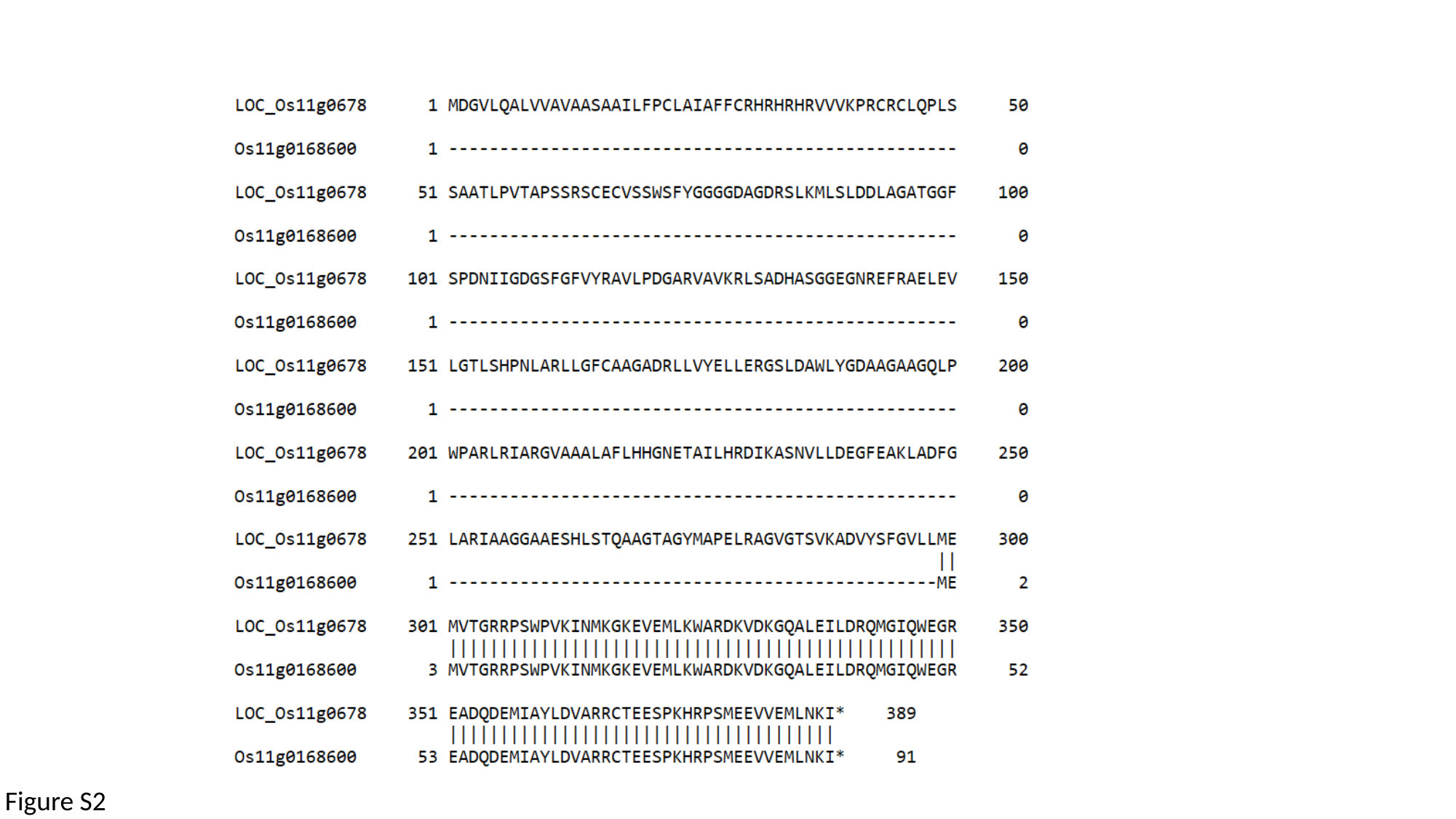

Figure S2

## Slide 3
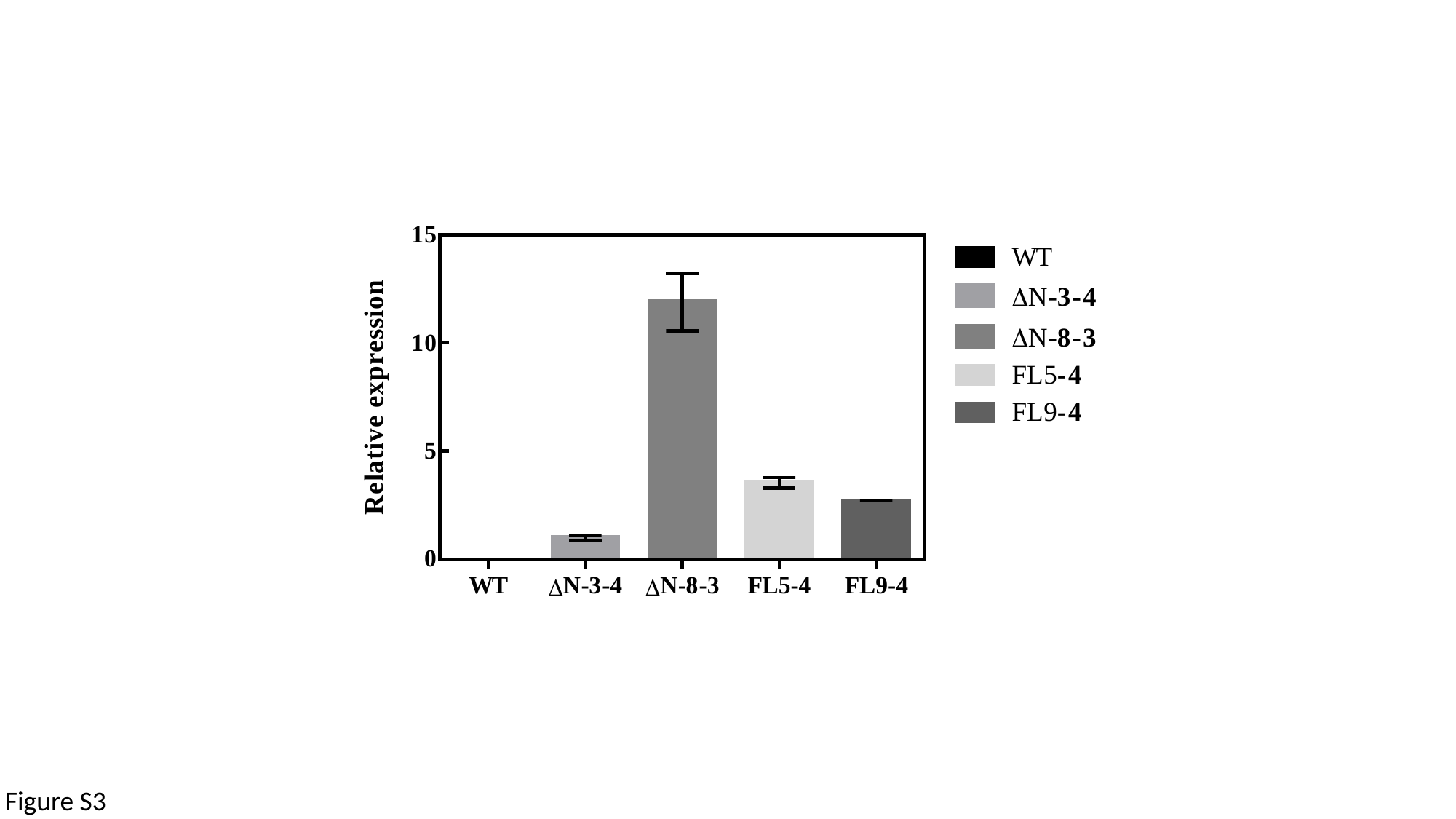

Figure S3

## Slide 4
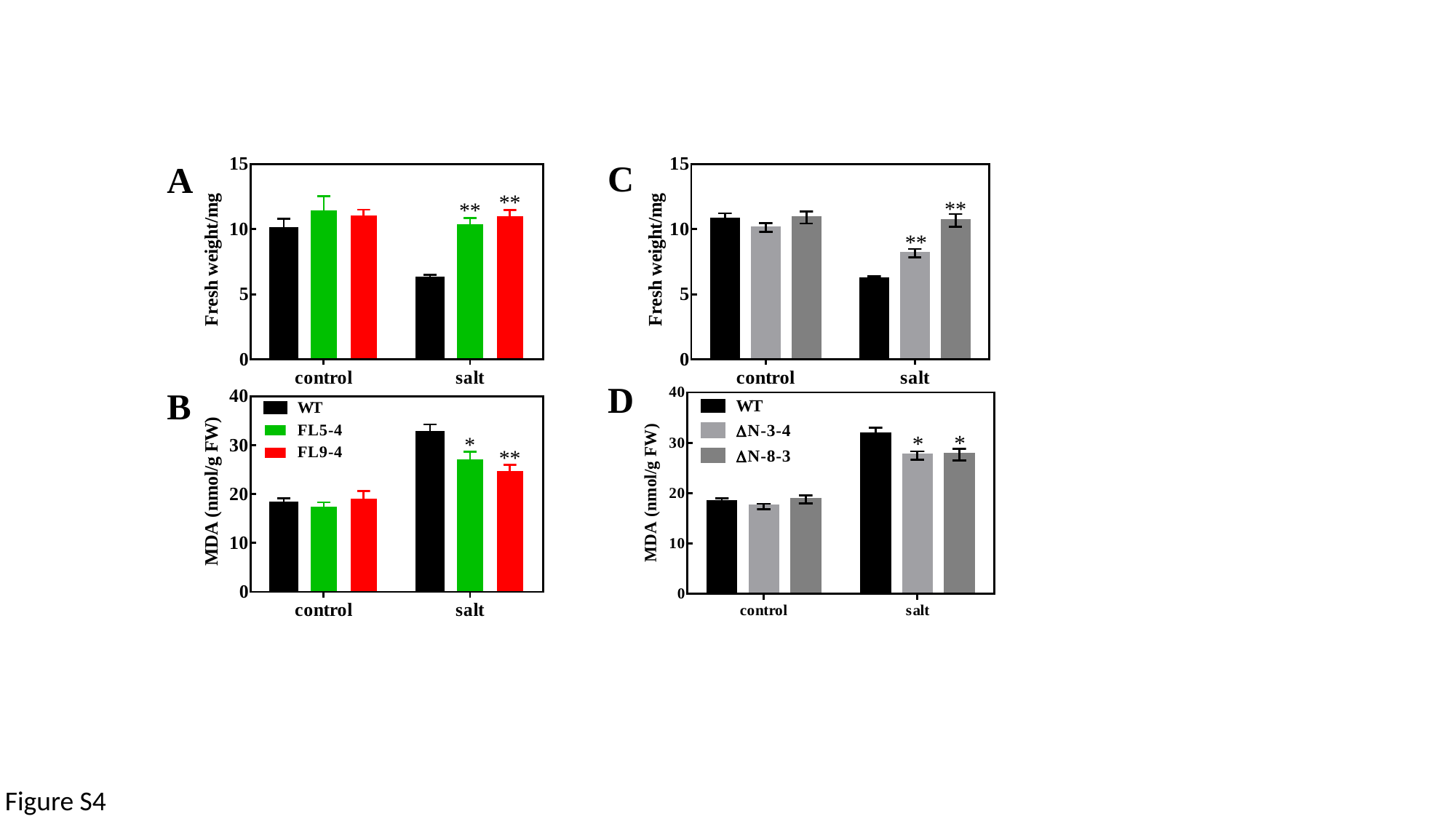

A
B
C
D
Figure S4

## Slide 5
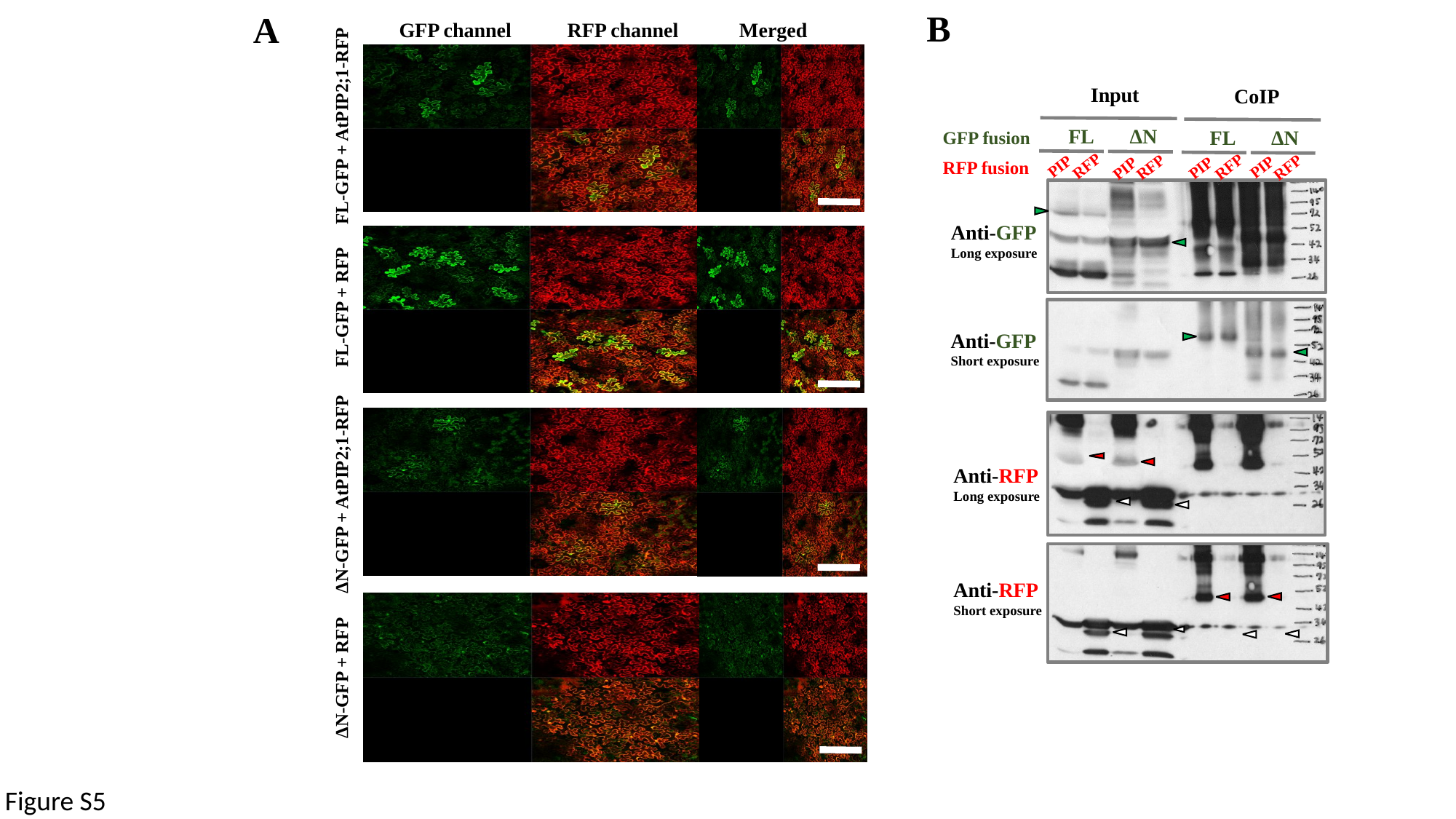

B
A
GFP channel RFP channel Merged
FL-GFP + AtPIP2;1-RFP
FL-GFP + RFP
ΔN-GFP + AtPIP2;1-RFP
ΔN-GFP + RFP
Input
CoIP
FL
ΔN
FL
ΔN
GFP fusion
RFP fusion
RFP
PIP
RFP
PIP
RFP
RFP
PIP
PIP
Anti-GFP
Long exposure
Anti-GFP
Short exposure
Anti-RFP
Long exposure
Anti-RFP
Short exposure
Figure S5

## Slide 6
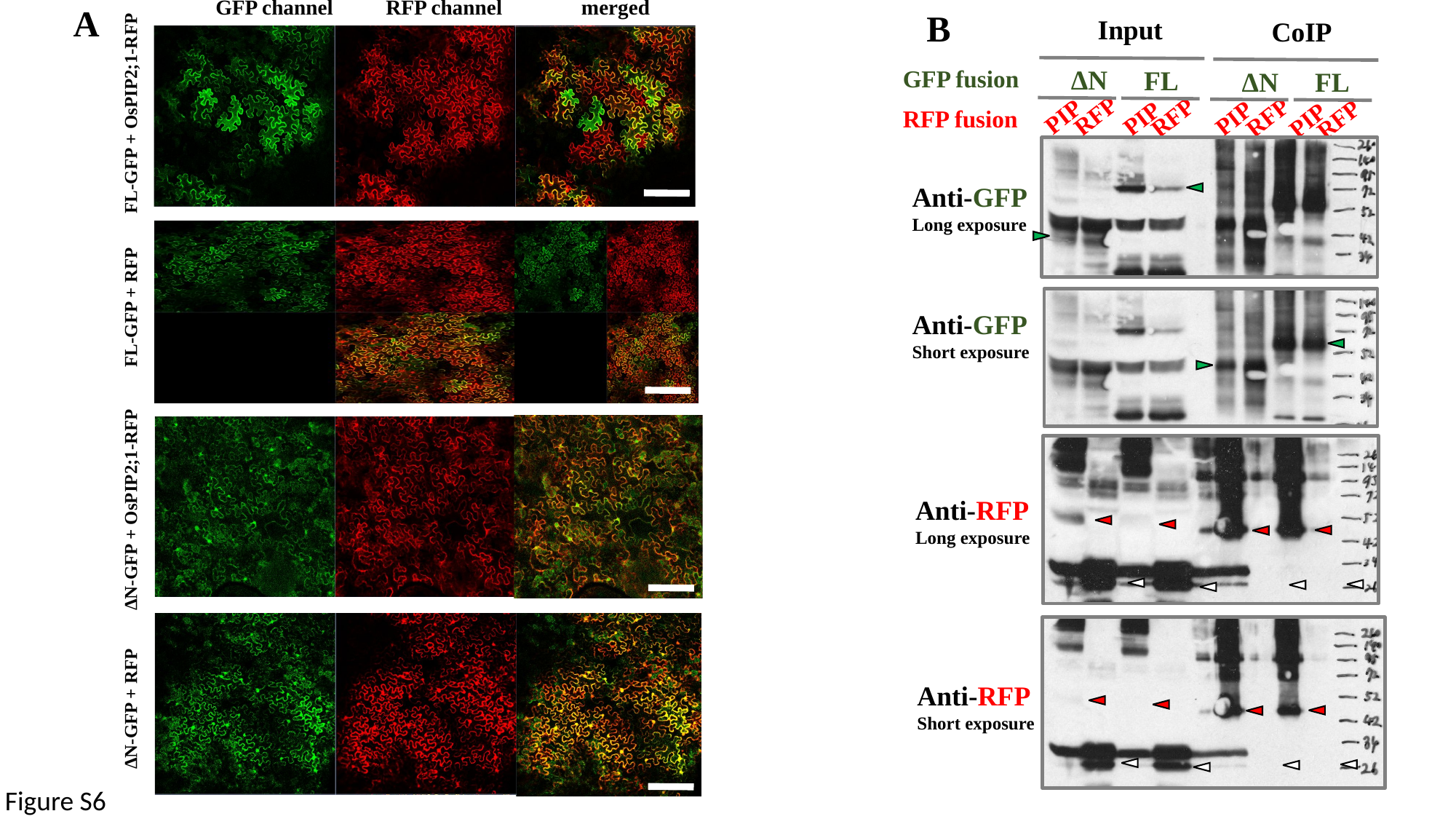

B
GFP channel RFP channel merged
FL-GFP + OsPIP2;1-RFP
FL-GFP + RFP
ΔN-GFP + OsPIP2;1-RFP
ΔN-GFP + RFP
A
Input
CoIP
ΔN
FL
GFP fusion
RFP fusion
ΔN
FL
RFP
PIP
RFP
RFP
PIP
PIP
RFP
PIP
Anti-GFP
Long exposure
Anti-GFP
Short exposure
Anti-RFP
Long exposure
Anti-RFP
Short exposure
Figure S6

## Slide 7
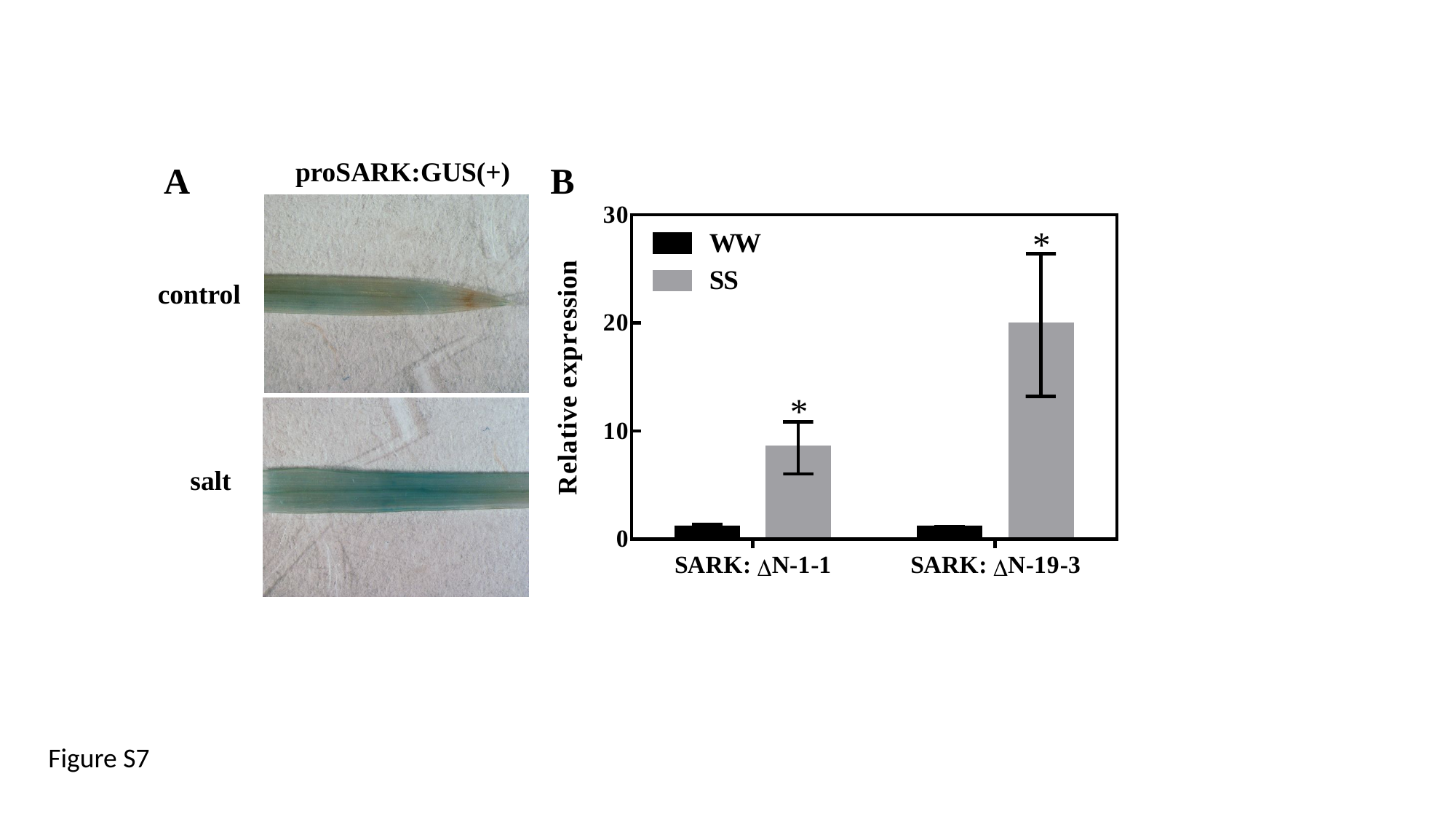

proSARK:GUS(+)
A
B
control
salt
Figure S7

## Slide 8
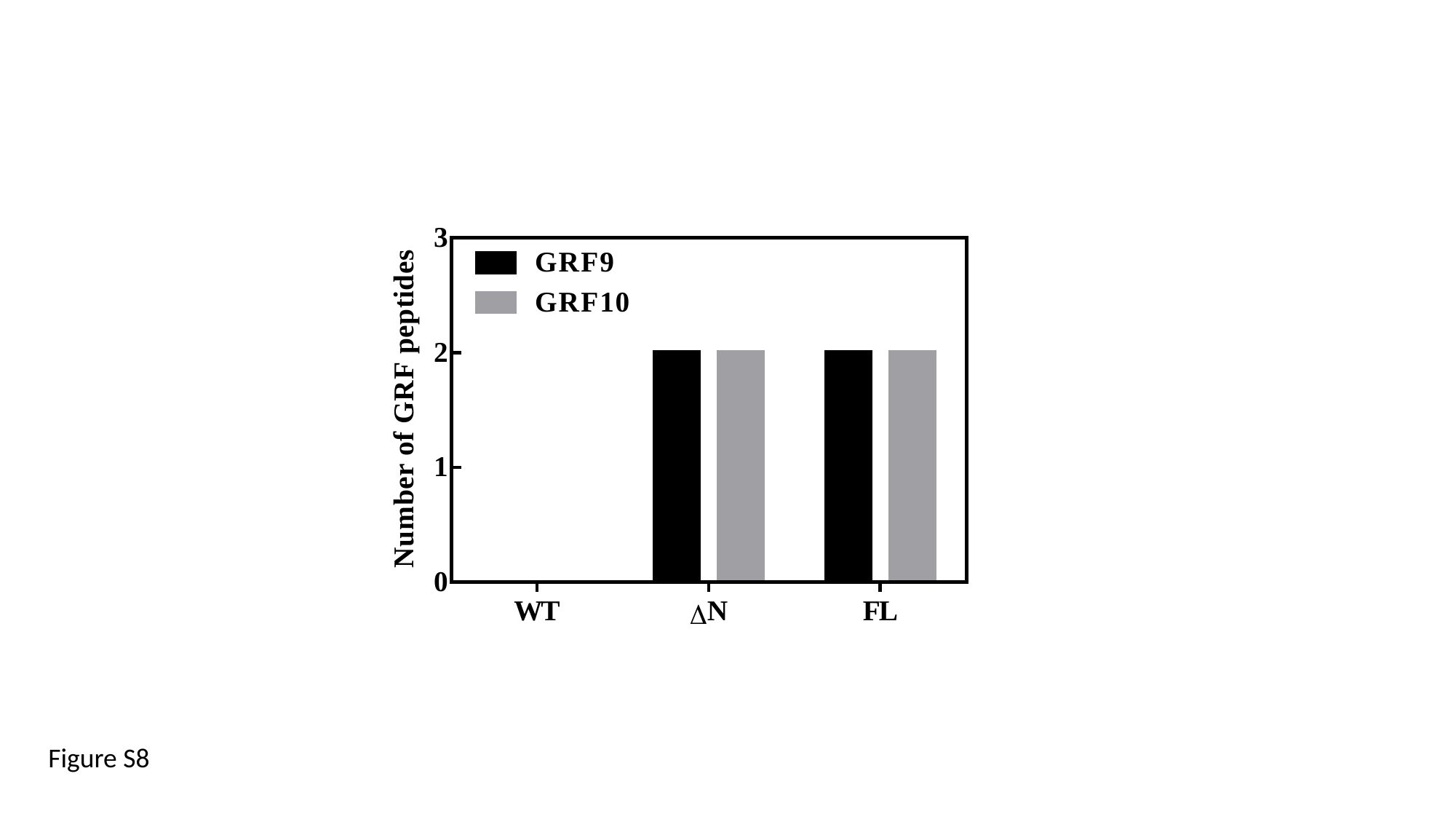

Figure S8
