# Supplementary material for: A Cytoplasmic Receptor-like Kinase Contributes to Salinity Tolerance
Source: Plants (Basel). 2020 Oct 17;9(10):1383. doi: 10.3390/plants9101383 (PMC7650656; doi:10.3390/plants9101383)
Supplement: Supplementary file 1 [file plants-09-01383-s001.zip › supplementary methods.docx]

**GUS staining**

Seeds of wild type *Brachypodium sylvaticum* and two transgenic lines were germinated on moist germination paper for 5 days at 4 °C in the dark. Young seedlings were then moved from the paper rolls to the growth chamber and grown at 26 °C /20 °C (day/night) 100 µmol m^-2^ s^-1^ light in 16h/8h day/night regime. Paper rolls with 20 d-old seedlings were exposed to 200mM NaCl for 24 h and stained using standard GUS protocols (Jefferson et al, 1987).

**Quantitative PCR analysis**

RNA was extracted from leaves of wild-type and transgenic *Brachypodium sylvaticum* plants under control and salinity conditions. First-strand cDNA synthesis, primer design, and quantitative PCR were performed according to the manufacturer’s procedures (SYBR^®^ Green RT-PCR Reagents Kit, Applied Biosynthesis, USA). Analysis of the relative gene expression was performed according to the comparative cycle threshold (2^−ΔΔCT^) method (Livak and Schmittgen, 2001). The housekeeping gene Ubiquitin 18 was used as the internal reference for *Brachypodium sylvaticum*  (Sade et al.,2018b) while the housekeeping gene TIP41 was used as the internal reference for *Arabidopsis* (Wang and Blumwald, 2014). Primers are given in Table S1.
