# Supplementary material for: A Cytoplasmic Receptor-like Kinase Contributes to Salinity Tolerance
Source: Plants (Basel). 2020 Oct 17;9(10):1383. doi: 10.3390/plants9101383 (PMC7650656; doi:10.3390/plants9101383)
Supplement: Supplementary file 1 [file plants-09-01383-s001.zip › Table S2.docx]

|  |  | Unique Peptides | Unique Peptides | Coverage  (%) | Unique Peptides | Coverage  (%) |
| --- | --- | --- | --- | --- | --- | --- |
| Protein | ID | WT | FL |  | ΔN |  |
| OsRLCK311 | BAIT | 0 | 4 | 42 | 3 | 42 |
| PIP2;1 | AT3G53420.1 | 0 | 3 | 20 | 2 | 8 |
| phospholipase D alpha 1 | AT3G15730.1 | 0 | 3 | 5.4 | 2 | 3.5 |
| GRF9 | AT2G42590.1 | 0 | 2 | 12 | 2 | 12 |
| GRF10 | AT1G22300.1 | 0 | 2 | 11 | 2 | 11 |
